# Editors’ Book Table

**Published:** 1874-02

**Authors:** 


### Editors' Book Table.

[NOTE.—All works reviewed in the columns of the CHICAGO MEDICAL JOURNAL may be found in the extensive stock of W. B. KEEN, COOKE & CO., whose catalogue of Medical Books will be sent to any address upon request.]

BOOKS RECEIVED.

*Homoculto logy*. An Essay. Published for the Author. For sale at the Progressive Publishing House, No. 24 Fourth Street, New York, and by all Booksellers.

The anonymous author may cry Eureka! he has found not only an idea, but a name for it; a panacea for "the thousand ills that human flesh is heir to;" a remedy, essentially radical, against the world, the flesh and the devil.

Arguing from the experience and practice of stock breeders, he proposes to improve the human race, by the elimination of all either physically, mentally or morally imperfect, by castration.

According to this long-eared philanthropist, convicted criminals, imperfectly developed children, the victims of disease, whether mental or physical, are all to be castrated, in order that the human race may be brought up by means of this process of elimination to the standard of "Homer, Byron, Socrates, Napoleon, Webster, Dickens, Dumas," etc. The author has been somewhat unfortunate in the selection of his models, we think, as some of the generations yet unborn might object to being cultivated up to blindness, lameness, cancer, etc., etc.

The thoughtful reader might like to be informed—the advantages of the system being conceded—by whom it was to be put into, and maintained in, practice. This is only suggested inferentially, on page 17, upon which the author condemns Mr. Peabody for the wrong done to *us*, in giving millions of pounds sterling to paupers, as, therewith being fed, they may "before long out-vote *us*."

*We*, then, are to be the executioners!

Further, we are told, "It will hardly be expected that (sic) in a preparatory discourse upon this great question, that (sic) a convincing and complete argument could be made. But little more will be attempted, therefore, than the announcement of the positions that will be hereafter maintained.

"1. Like begets like—the good and the bad elements of humanity repeat themselves.

"2. Improvement can only be made by breeding, by careful selection, purity of blood, stock, ancestry, and the philosophy of procreation.

"3. The fountains of mental, moral and physical diseases must be closed; there must be no duplicates of physical, mental or moral incurables in the next generation."

In reference to the first proposition, with a full appreciation of the character of the "Essay" before us, we can only say "'tis true, 'tis pity; and pity 'tis, 'tis true."

The second is scarcely original, except "the philosophy of procreation," which we suppose means an appreciation of the advantages of castration.

It is to be hoped that the author will demonstrate the practicability of his third proposition by a personal application of his system, or accomplish it still more effectually *more Japonico*, by hari-kari. H.

*Expert Testimony.* By THAD. M. STEVENS, M.D. From the *Indiana Journal of Medicine* for October, 1873.

The objective point of this essay appears to be the appointment of State, or District, Boards of Experts, to testify concerning medico-legal questions, and also to secure their adequate compensation.

We say, appears to be, as the paper is somewhat difficult to comprehend, by reason of the singular obscurity of its style, and the disregard of orthography, etymology and syntax manifested by the writer, who has "*thought* it best to offer some *thoughts*"; who talks of "*Socialogy*," and says that "some notorious *examples* of the incompetency of medical testimony *exists*"; that "the most thorough and conclusive chemical examination could not make *headway* or *dispell* the prejudice engendered by such faulty testimony"; who prescribes "one-fifth or one-fourth gr. of morphine and bromide *potasium*," leaving us somewhat doubtful as to drug and dose—but tells us that "the *results* of the post mortem *was* of great importance." Further, we learn that "there is a certain *tactus erudicus*," etc.; and later, that "it becomes us all to look after this *phrase* of the matter," and "*apropo* of this"—

Since "we must keep in mind the difference between *common* witnesses and experts," we are puzzled about the status of the writer; is he an expert? he certainly is a most uncommon witness.

H.

*An Essay on the Principles of Mental Hygiene.* By D. A. Gorton, M.D. Philadelphia: J. B. Lippincott & Co. 1873.

Says our author, in his preface, "He who reads the following pages with the attention which the importance of the subject demands, cannot fail to remark the number of quotations introduced from the writings of distinguished savants, past and present. To my mind, that is the best feature of my volume." An honest confession, and certainly true; they constitute not only the best, but by far the *larger* portion of the volume; indeed, without these quotations there would have been left but the cement,serving to join, not unite, the various pieces of this literary mosaic, and possessing about the same relative value thereto as the paste in which they are imbedded to the brilliant mosaics of the Vatican.

In the concluding paragraph of the preface, the author expresses his deep sensibility to "the shortcomings and imperfections of the volume," and also the consciousness "of having done the best I could with the leisure and materials at my command."

In regard to which we must express our conviction that the obligation to publish a book rests upon no man, and least of all when he has neither time nor material for his work.

The body of the volume is divided into six chapters, from which we, following the author's example, will make a few quotations. Referring to the mental influence of physical agents, he says: "An unsightly physical deformity represents so much morbid force spent in the direction of the least harm to the mental character of the individual. A goitre, for example, may, and I believe often does, hold in its cells the condensed essence of a mania," etc., etc. Now, if it be true, as the author has said on the preceding page, that "the phenomena of nature proceed entirely from legitimate antecedents," we fail to appreciate his *morbid force*, and if "an unsightly physical deformity represents so much morbid force spent in the direction of the least harm to the mental character of the individual," how does it happen that all human experience has taught the constant association of idiocy and physical deformity? and where the consistency of the Horatian maxim that adorns his title page—"*Sana mens in corpore sano*"? (a false construction.)

In the fourth chapter, on moral and religious influences, the author tells us, "Long ago, in our youthful days, we 'experienced religion,'" etc., and a little further on he says, "true religion is not in the market, and cannot be bought and sold, bartered for *nor begged* with a *prayer*"; from which we may form some idea of the character of that religion which he "experienced"; in further witness to which hear him—"While, therefore, fear, ignorance and credulity are the foundation of superstition, in its modern acceptation, superstition and theology are the foundation of religion;" probably, of that which he "experienced," minus the theology.

Again, he says, "The Spiritual Head of the Church assured his followers from time to time that the secret of so-called miracle-working was credulity: 'And Jesus said unto them, *Believe* ye that I am able to do this? They said unto him, Yea, Lord. Then touched he their eyes, saying, According to your *faith* be it unto you;' and to the woman who had been ill for twelve years, and who was finally cured by a single touch of his garment, he said: 'Daughter, be of good comfort; thy faith hath made thee whole,' etc., etc. Such is the foundation of the absurd pretensions of all the great religions of the world; witchcraft; fortune-telling; magnetism; medical charlatanry; and also of other curious psychological phenomena, of which the world is so full to-day."

Concerning marriage, the author, quoting Mill—an authority hardly orthodox upon this subject—says, "it is hardly possible for one who is in these bonds to attain exalted virtue," or "to retain it if once acquired." And again, "It would be a very unhappy alternative, to be sure; but personal character of a high order would more than compensate one for the conventional odium of bastardy." To which we can only reply, chacun a son gout. "Bastards, while they are conceived in sin, are generally bred and reared in virtue." How this singular inconsistency occurs we are not informed. But basta! enough! to review the book upon its own merits is difficult, as there is very little of it, and that little has very little merit. The author is evidently a reader, extensive, certainly, but hardly comprehensive, who has served up a sort of literary ragout in which the multiplicity of imported flavors are scarcely sufficient to disguise the watery decoction of false philosophy, infidelity and blasphemy in which they are suspended.

H.

*A Handbook of the Theory and Practice of Medicine.* By FREDERICK T. ROBERTS, M.D., B.Sc., M.R.C.P., etc., etc. Philadelphia: Lindsay & Blakiston. 1874.

There will be very few of our readers, we think, who, having read Dr. Roberts' "Handbook," will be willing to spare it for any length of time from their office tables. If there is a book in the whole of medical literature in which so much is said in so few words, it has never come within our reach. So clear, terse and pointed is the style, so accurate the diction, and so varied the matter of this book, that it is almost a dictionary of practical medicine.

We have examined it carefully for defects, and confess that the search was fruitless. We have looked for new remedies, and found all that we sought and more that we wot not of. We have sought for the results of recent investigations, and found that the author had anticipated our search.

Without going into a detailed analysis of the work, we can conscientiously say that we scarcely know one destined to prove of more practical utility to students and general practitioners, whose studies it will greatly facilitate, or to the expert, even, who will find it a most valuable reminder in any department of our art. It is emphatically a book of ideas rather than words, and is moreover intensely practical. After having found it necessary to condemn certain volumes so earnestly, it is exceedingly gratifying to turn to one like this which we can most heartily praise. H.

*Mind and Matter;* or, Psychological Inquiries, in a series of Essays, intended to illustrate the Mutual Relations of the Physical Organization and the Mental Faculties. By SIR BENJAMIN BRODIE, Bart., D.C.L., Vice-President of the Royal Society. With additional notes by an American Editor. New York: William Wood & Co., 27 Great Jones St. 1873.

The admirable dialogues of Eubulus, Crites and Ergates are so well known to American students through the edition already published by the Messrs. Wood & Co., that criticism seems superfluous.

We are glad, hovever, to welcome this new edition, as it will serve to attract the attention of the younger members of the medical profession to a work which cannot fail to benefit its careful reader. H.

*An Introduction to Practical Chemistry.* By JNO. E. BOWMAN, F.C.S., Late Professor of Practical Chemistry in Kings College, London. Edited by CHARLES L. BLOXAM, F.C.S., etc., etc. Sixth American, from the sixth and revised English edition. Philadelphia: Henry C. Lea. 1873.

A book which has passed through six editions on each side of the ocean needs no certificate of popularity. One who studies it will perceive that its popularity is based upon its intrinsic merit. Written originally as a text book for the chemistry class of King's College, London, it was adapted especially to the wants of those just entering upon a course of chemical studies, but will be found sufficiently comprehensive to fulfill the requirements of the large majority of general practitioners, whose chemical acquirements rarely extend beyond its limits. It possesses an especial advantage in its small size, the author having been, seemingly, careful to avoid the dilution of his matter, and the expansion of its bulk by useless verbiage, from which it is singularly free. The instructions are clear and explicit, and the illustrations which accompany them well designed and executed. H.

*Lectures on Diseases and Injuries of the Ear.* Delivered at St. George's Hospital. By W. B. DALBY, F.R.C.S., M.B., Cantab., Aural Surgeon to the Hospital. With twenty-one illustrations. Philadelphia: Lindsay & Blakiston. 1873.

These lectures constitute a useful little manual of some of the diseases and injuries of the ear, more especially of the external and middle ear. The work lacks order, somewhat, the subjects being considered rather from basis of the relative anatomy of the organ. It is scarcely adapted to students, as it takes for granted the possession by the reader of a knowledge of the anatomy and physiology of the ear, as without such knowledge he would be greatly embarrassed in the study of the work.

To practitioners, somewhat familiar with the mechanism and functions of the auditory apparatus, the book will prove serviceable for reference, the subjects which it comprises being presented clearly and practically, by an author thoroughly familiar therewith. The concluding pages are occupied by a description of the different methods of educating deaf mutes, by "Dactylology," and by "Lip-Reading," which possesses much interest. H.

*The Leprous Diseases of the Eye.* With six colored plates. By Dr. OLE B. BULL, Member of the Norwegian Medical Society and Private Docent in Ophthalmology at Christiana; and Dr. G. A. HANSEN, Member of the Norwegian Medical Society and Physician to the Leprosy Hospital at Bergen. Christiana: Published by Albert Cammermyer. 1873.

The above treatise possesses much interest for ophthalmologists, if the authors' expressed opinion be correct, "that there is no disease which so frequently gives rise to disorders of the eye, as leprosy does," and by "leprosy" they designate elephantiasis, or lepra tuberculosa, and eleps, or lepra levis. The fact, to which they allude, that the consideration of this class of diseases has been omitted from ophthalmological literature, seems opposed to their opinion.

However this may be regarding the world at large, it is doubtless true in Norway, where leprosy prevails so extensively, as the excellent descriptions of the different forms of the disease, and the thorough familiarity with them, displayed by the authors, could only result from the patient study of a large number of cases, and this would be possible only where the disease was of frequent occurrence. It is quite possible, moreover, that this disease may be imported into our own country, more especially the north-western portions of it, which are becoming the homes of large numbers of Norwegian immigrants, and may become indigenous amongst them.

The style of the work is excellent, and although the translator is not referred to, its linguistic accuracy proves that this portion of the work has been accomplished by one equally expert in English as in Norwegian. H.

BOOKS, PERIODICALS AND PAMPHLETS

RECEIVED SINCE PUBLICATION OF LAST NO.

*Philadelphia Medical Times*—Dec. 13, 20, 27, 1873.

*Medical Record*—New York, Dec. 15, 1873.

*The Medical Press and Circular*—London, Nov. 26, 1873.
*The Clinic*—Cincinnati, Dec. 20 and 27, 1873.
*The London Lancet*—Dec., 1873.
*The Richmond and Louisville Medical Journal*—Dec., 1873.
*The Boston Medical and Surgical Journal*—Dec., 1873.
*The Cincinnati Lancet and Observer*—Dec., 1873.
*The Medical and Surgical Reporter*—Philadelphia, Dec. 13, 20, 27, 1873, and Jan. 3, 1874.
*Atlanta Medical and Surgical Journal*—Nov. and Dec., 1873.
*The Pacific Medical and Surgical Journal*—Dec., 1873.
*The Indiana Journal of Medicine*—Dec., 1873.
*The Virginia Clinical Record*—Dec., 1873.
*The Dental Cosmos*—Philadelphia, Dec., 1873.
*The Ohio Medical and Surgical Reporter*—Nov., 1873.
*The Medical Herald*—Leavenworth, Kansas, Dec., 1873.
*The Leprous Diseases of the Eye.* With six colored plates. By DR. O. B. BULL and DR. G. A. HANSEN, Members of the Norwegian Medical Society, etc., etc., Christiana and Bergen. Christiana: Albert Cammermeyer. 1873.
*The Physician and Surgeon*—Baltimore, Dec., 1873.
*The Medical Examiner*—Chicago, Dec. 15, 1873, and Jan. 1, 1874.
*Report of the Health Officer* of the City and County of San Francisco, Cal., for fiscal year ending June 30, 1873.
*Ninety-First Annual Catalogue* of the Medical School of Harvard University.
*The Fourth, Fifth and Sixth Annual Reports* of the Central Free Dispensary of West Chicago, to June 30, 1873.
*Le Progres Medical.* Paris, No. 16, 17, 18, 19, 20, 21, 1873.
*The Canada Medical Record*—Dec., 1873.
*The Practitioner*—Dec., 1873.
*Ohio Medical and Surgical Reporter*—Nov., 1873.
*Homocultology:* An Essay.
*Expert Testimony.*
*Evidences of Life in the Newly Delivered Child.* By WM. B. ATKINSON.
*Medical Examiner*—Jan. 1, 1874.
*Medical Herald*—Leavenworth, Kansas, Jan., 1874.
*Philadelphia Medical Times*—Jan. 3, 1874.
*New York Medical Record*—Jan., 1874.
*Granular Cell found in Ovarian Fluid.* THOS. M. DRYSDALE Collins, Publisher, Phila.

*Toner Lectures.* Lecture 1 : The Structure of Cancerous Tumors, etc. J. J. WOODWARD, M.D., U. S. A.
*Nebraska State Society, Proceedings of,* 1873.
*Arkansas Medical Association, Proceedings of,* 1873.
*Washington Territory, Medical Society of, Proceedings of,* 1873.
*St. Louis Medical and Surgical Journal*—Jan., 1874.
*Canada Medical and Surgical Journal*—Dec., 1873.
*Kansas City Medical Journal*—Dec., 1873.
*Clinic*—Jan. 3, 1874.
*Practitioner*—Dec., 1873.
*London Medical Record*—Nov., 1873.

ANNOUNCEMENTS.

Geo. P. Putnam's Sons, New York, have in preparation, a translation by Doctors M. D. Mann and S. B. St. John, of a recent work by Professor Ludwig Buhl, of Munich, on Inflammation of the Lungs, Tuberculosis, and Consumption.

Dr. Geo. M. Beard, of New York, proposes to found "a journal devoted to the Special Departments of Electricity, in its relations to Medicine and Diseases of the Nervous System," under the title "Archives of Electrology and Neurology," to be issued semi-annually, commencing early in 1874.

Doctors Jewell and Bannister, of Evanston, announce their intention to issue early in the present year, a quarterly, to be entitled "The Chicago Journal of Nervous and Mental Disease." Its scope is designed to be comprehensive, and its range extensive. It has our best wishes for its success and prosperity.

Messrs. Keen and Cooke have now in press a translation of Dr. Arthius' work, on the Application of Static Electricity to the Treatment of Nervous and Rheumatic Affections, by J. H. Etheridge, Professor of Materia Medica Rush Medical College. The original work possesses much interest, which will in no wise be diminished in its translation by the facile pen of so accomplished a scholar as Prof. Etheridge.

